# A Large Alveolar Abscess

**Published:** 1867-04

**Authors:** Gam’l Jackson

**Affiliations:** Winona, Minn


### A LARGE ALVEOLAR ABSCESS.

BY DR. GAM'L JACKSON, WINONA, MINN.

Miss ——, aged twenty years, of scrofulous habit, came to my office in January last to have some roots of teeth extracted. After removing those of the second superior molar of the left side, the patient said she felt a throbbing pain there. The adjoining wisdom tooth was decayed on the anterior side, and suspecting its roots to be diseased, I decided to remove it. I was surprised at the slight force required to luxate it, and was further surprised that so loose a tooth should still resist an apparently adequate effort to remove it. An examination disclosed that the tooth was still firmly invested by the process, and that the whole was held in place only by the attachments of the surrounding soft tissue. These were dissected off, when the entire investing process of the tooth, extending forward as far as the alveoli of the adjacent molar, and three-quarters of an inch upward, together with a cyst measuring at first seven-eighths of an inch by three-fourths of an inch in diameter, came away with the tooth. The cyst was egg-shaped, and occupied a horizontal position over the second and third molars, the small end pointing downward and forward. Its side was evidently penetrated by, and perhaps connected with the roots of the second molar, and it was further attached to the periosteum of the anterior buccal root of the third molar. The posterior root had not been reached by the encroaching lesion, but the other two were shortened about one-third of their length, thus forming a part of the smooth floor of the concavity occupied by the cyst. The abscess had entirely cut off the above described portion of bone equally on the palatal and buccal sides, the only remaining attachments being a thin strip at the alveoli of the second molar, and a similar one at its posterior perpendicular extremity, and these were so slight as to have been readily broken by the fingers alone.

The patient lives at a distance from this city, and I have not had an opportunity to examine the case since.

